# Studies on Radiation Damage to Improve the Efficiency and Data Quality

**DOI:** 10.1063/4.0001161

**Published:** 2025-10-27

**Authors:** Palani Kandavelu, Zhongmin Jin, Zheng-Qing "Albert" Fu, John Chrzas, John P. Rose, Bi-Cheng Wang

**Affiliations:** SER- CAT/University of Georgia, Athens, GA, USA

## Abstract

The high levels of flux at a fourth-generation synchrotron are shown to have significant beam heating effects with increasing risk of radiation damage X-ray crystallography technique is widely used to determine the three-dimensional structures of macromolecules and it is very important to know and how it might affect an X- ray diffraction experiments and the resulting structures. There are two types of radiation damages (global and specific).The radiation damage process is dose dependent and there is no technique available to prevent. X- ray crystallography can be used to monitor the damages by collecting consecutive data sets in the same protein crystal to track the progression of damages. Damage incurred during data collection in macromolecular crystallography (MX) limits the information that can be obtained from a single crystal, and it may also prevent getting the solution of structure. Each year, hundreds of scientists are using SER-CAT to conduct X-ray diffraction experiments many of which are directly related to human diseases. Their efficiency and quality of the data collected for various high impact scientific projects will be depending upon the how precisely we study this radiation damage and provide a general data collection strategy especially for the increased intensity (after APS-U) to SER-CAT user community. To study the consequences and progression of radiation damages, 10 consecutive datasets were collected using single trypsin crystal and analyzed. The results for both global and specific damage effects will be presented.

